# Miscellaneous Medical Intelligence

**Published:** 1851-05

**Authors:** 


ARTICLE IV.

### MISCELLANEOUS MEDICAL INTELLIGENCE.

Dr. John Curwen, late assistant physician in the Pennsylvania Hospital for the Insane, has been appointed Superintendent of the Pennsylvania State Lunatic Asylum at Harrisburg.

Prof. Thos. Reyburn, of the University of St. Louis has resigned his chair in that institution.

Not having received the January number of the Ohio Med. and Surg. Journal, we were not aware of the editorial change in that periodical. Prof. R. L. Howard, has assumed the laborious and responsible position vacated by Dr. Smith. His labors will be satisfactory to the readers of that work, if they appreciate correct taste and sound judgment.

We observe by the tariff of fees adopted by the Medical profession of Richmond, Va., that they charge from $25 to $50 for making a post mortem examination in cases of legal investigation. The ordinary obstetrical fee is $20.

The *Northern Lancet and Gazette of Legal Medicine*, published at Plattsburg, N. Y., comes to us in a new and greatly improved form. It has been reporting a series of Lectures on Medical Jurisprudence, by Anthony Todd Thompson, which are exceedingly interesting.

Prof. Blaney has recently successfully treated a case of poisoning by arsenic, where a large quantity of the poison had been taken by a child, with Calcined Magnesia and Hydrated per Oxide of Iron. The case will be reported.

Prof. Thos. Spencer, of Milwaukee, Wis., who was elected Emeritus Prof. of Theory and Practice of Medicine in Rush Medical College last fall, after resigning his chair in that Institution on account of ill health, we are pleased to learn has very much improved since that time, and now has a fair prospect of entire recovery.

Professors Jackson and Bigelow, of Boston, are on a visit to Europe. A large number of physicians have gone over. No doubt the great London World's Fair has influenced many to go the present year, who designed to visit Europe within a few years of this time.

Dr. Cain, one of the Editors of the Charleston Medical Journal, has recently been using the inunction for scarletina as recommended by Dr. Schneeman, with the most flattering results. Dr. Ebert, of Berlin, N.Y., reports 28 cases of the same treatment, in the N.Y. Med. Jour., with the most satisfactory success.

The Medical College of Ohio, is about to erect a new college edifice in Cincinnati.

The New Jersey Medical Reporter, is changed from being a quarterly to a monthly Journal. This denotes prosperity, which it richly deserves.

Professors Brainard and Davis have gone to Charleston, S. C., to attend the meeting of the American Medical Association, and Professors Blaney and Evans to Cincinnati, to attend the meeting of the American Association for the advancement of Science; we shall therefore be able to furnish our readers a synopsis of the proceedings of both these bodies at an early day.

Small Pox has been quite prevalent in Chicago for a few months past.

We hear that there were over sixty students in attendance on the first course of lectures in the Medical Department of the University of Michigan. Truly an auspicious beginning—success attend the Institution.

Some of the advocates of high charges for lecture fees in Medical Schools, think it not dishonorable for a richly endowed institution to lecture free; but if those who are not rich, in the immediate vicinity of them, adopt the same policy, it is highly dishonorable. We long ago learned that wealth secured the homage of many persons, but did not know that it absolved allegiance to any rule of propriety.

Dr. R. D. Arnold, of Savanah, Geo., has been presented with a splendid piece of plate as a token of respect for his gratuitous and faithful services, as physician to the Hospital for a term of 15 years. It bears the appropriate inscription, "I was sick, and ye visited me."

The Medical Department of the University of Nashville, has been organized. The first course of lectures will commence in November next.

It would appear that Iowa has actually levied a tariff on Medical knowledge, and gives the graduates of the Keokuk school in that State "important legal immunities" over those who get their education out of the State. Those connected with that school think it fine fun; but we doubt their being able to make students attend their school, even by a resort to law. The "*immunities*" would'nt pay.

Tannin applied to the inflamed part and pressed down under the edge of the nail, has been found good for inverted toe nail, by Dr. Drake, of Lexington, Ky. He kept the application on with a roller bandage.

M. Rigal has communicated to the Surgical Society of Paris the discovery that if a pistol be discharged with the muzzle held tightly against the chest, so as to prevent the ingress of air into the barrel, the ball will not penetrate the wall of the thorax but be deflected at a considerable angle. Better not try it.

The Boston Medical and Surgical Journal says in reference to Prof. Davis' History—

"Dr. Davis, in this little volume, has given us a history of the rise and progress of medicine, medical institutions, &c., in this country, from the landing of the Pilgrims at Plymouth, in 1620. Such reviews of the past, as Dr. Davis justly remarks, are not only peculiarly appropriate, but in the highest degree profitable to the members of our Profession. It is also pleasant to compare the dogmas of the profession in past times, with the present advanced state of medical science. Yet there were many master spirits then in the medical ranks, who labored with a zeal worthy the imitation of their successors. Dr. Davis, in mentioning the wants of the profession of the present day, assumes the proper position; and if his suggestions should be adopted, the greatest practical good would follow, both as regards the interests of the profession and the welfare of individuals generally.

